# Childhood cognitive ability and adult mental health in the British 1946 birth cohort

**DOI:** 10.1016/j.socscimed.2007.02.027

**Published:** 2007-06

**Authors:** Stephani L. Hatch, Peter B. Jones, Diana Kuh, Rebecca Hardy, Michael E.J. Wadsworth, Marcus Richards

**Affiliations:** a Columbia University, New York, NY, USA; b University of Cambridge, UK; c MRC National Survey of Health and Development, University College London, UK

**Keywords:** UK, Adult mental health, Prospective cohort, Childhood cognitive ability, Educational attainment, Gender

## Abstract

We examined whether childhood cognitive ability was associated with two mental health outcomes at age 53 years: the 28 item General Health Questionnaire (GHQ-28) as a measure of internalising symptoms of anxiety and depression, and the CAGE screen for potential alcohol abuse as an externalising disorder. A total of 1875 participants were included from the Medical Research Council National Survey of Health and Development, also known as the British 1946 birth cohort. The results indicated that higher childhood cognitive ability was associated with reporting fewer symptoms of anxiety and depression GHQ-28 scores in women, and increased risk of potential alcohol abuse in both men and women. Results were adjusted for educational attainment, early socioeconomic status (SES) and adverse circumstances, and adult SES, adverse circumstances, and negative health behaviours. After adjusting for childhood cognitive ability, greater educational attainment was associated with reporting greater symptoms of anxiety and depression on the GHQ-28. Although undoubtedly interrelated, our evidence on the diverging effects of childhood cognitive ability and educational attainment on anxiety and depression in mid-adulthood highlights the need for the two to be considered independently. While higher childhood cognitive ability is associated with fewer internalising symptoms of anxiety and depression in women, it places both men and women at higher risk for potential alcohol abuse. Further research is needed to examine possible psychosocial mechanisms that may be associated with both higher childhood cognitive ability and greater risk for alcohol abuse. In addition, the underlying mechanisms responsible for the gender-specific link between childhood cognitive ability and the risk of experiencing internalising disorders in mid-adulthood warrants further consideration.

## Introduction

Studies have reported an association between low cognitive ability in childhood (Batty, Deary, & Macintyre, 2006; Batty, Mortensen, & Osler, 2005; Feinstein & Bynner, 2004; van Os, Jones, Lewis, Wadsworth, & Murray, 1997) or early adulthood (Zammit et al., 2004), and adverse mental health outcomes in mid adulthood. While reasons for this association are not fully understood, a number of dynamically interactive processes may be involved. The present study aimed to examine several mediating variables involved in these processes that might account for the link.

### Cognitive ability and mental health status: proposed mechanisms

Cognition may be indirectly associated with mental health, to the extent that it is an indicator of “system integrity” (Whalley & Deary, 2001), i.e. integrity of the nervous system, or of its underlying physiological processes that influence mental health. These processes include the hypothalamic–pituitary–adrenal (HPA) axis, the growth hormone axis, and the thyroid system (McEwen, 2003; Thompson, Syddall, Rodin, Osmond, & Barker, 2001; Welberg & Seckl, 2001), all of which also influence cognitive function. Indeed, stress-responsive endocrine systems such as the HPA axis provide a plausible mechanism for the common effect of early adverse experiences on cognition and mental health, since over-activation of these systems damages neural systems supporting learning and memory and emotional responsivity (Sapolski, Krey, & McEwen, 1986; Sherwood Brown, Varghese, & McEwen, 2004; Welberg & Seckl, 2001). These adverse experiences can include, but are not limited to, institutional privation (Rutter, Kreppner, & O’Connor, 2001), parental divorce (Fergusson, Lynsky, & Horwood, 1994; Richards & Wadsworth, 2004; Rodgers, 1990), and poor parenting (Guo & Harris, 2000; Richards & Wadsworth, 2004; Rodgers, 1990, 1996).

Cognition also may be associated with mental health because it may be part of a causal chain that modifies mental health risk. Cognitive development partly determines educational attainment, social and occupational status, and income (Feinstein & Bynner, 2004; Kuh, Head, Hardy, & Wadsworth, 1997; Neisser et al., 1996; Richards & Sacker, 2003). Socio-economic status (SES), in turn, influences exposure to stress from a range of sources (Dohrenwend & Dohrenwend, 1974; Pearlin, 1989; Pearlin, Menaghan, Lieberman, & Mullan, 1981; Turner, Wheaton, & Lloyd, 1995), which may ultimately affect mental health. For example, persistent stressors may result from sustained financial hardship (Lynch, Kaplan, & Shema, 1997) and psychosocial strains from marital, occupational and parenting roles, particularly in the context of limited social support.

### Other key factors impacting adult mental health outcomes

Similar to the processes proposed for cognitive ability, education has the potential to lead individuals to environments or behaviours that protect health. Education has been suggested to mediate the effects of early life adversity or material and socio-cultural resources from the family on mental health (Blane, 2003); to operate as a fundamental social cause of status and health inequalities by directly shaping access to resources such as money, knowledge, power, prestige (Link & Phelan, 1995); and to protect against exposure to negative life events and chronic stressors (Pearlin, 1989). Educational attainment also provides the skills and qualifications that structure other components of SES, such as occupational status and income (O’Rand, 2001; Mirowsky & Ross, 2003). Generally, the impact of education on health is present throughout adulthood (Miech & Shanahan, 2000) and higher educational attainment is associated with better mental health outcomes (Araya, Lewis, Rojas, & Fritsch, 2003; Dohrenwend et al., 1992; Lorant et al., 2003).

In contrast, a range of evidence suggests that early adversity is associated with poor adult mental health outcomes (Brown & Harris, 1978; Kessler & Magee, 1993; Turner & Lloyd, 1995). Among adult adverse circumstances, unemployment and financial hardship are generally related to childhood cognitive ability (Fergusson, Horwood, & Ridder, 2005) and poor mental health (Dooley, Prause, & Ham-Rowbottom, 2000; Mirowsky & Ross, 2001; Pearlin et al., 1981).

Health behaviours, such as exercise and cigarette smoking, may influence risk of poor mental health, but more importantly may operate as coping mechanisms in response to a negative outcome, thereby possibly obscuring the true effect size of an adverse exposure. Several studies document the benefit of exercise in reducing symptoms of anxiety and depression (Craft & Landers, 1998). Whereas, the cessation of cigarette smoking is associated with higher childhood cognitive ability (Taylor et al., 2003) and active cigarette smoking is associated with depression (Duncan & Rees, 2005).

### The British 1946 birth cohort

The Medical Research Council National Survey of Health and Development (NSHD; the British 1946 birth cohort) provides an opportunity to investigate these issues within a comprehensive analytical framework. Previous work in this cohort has demonstrated an association between childhood cognitive ability and affective disorder at either 36 or 43 years (van Os et al., 1997), independently of childhood onset affective disorder. However, this association was only adjusted for gender and social class, and cannot therefore elaborate causal pathways.

Concerning externalising symptoms, while Richards, Hardy, and Wadsworth (2005) reported effects of alcohol consumption on midlife cognitive change in the NSHD, no study to date has investigated associations between childhood cognition and potential alcohol abuse in this cohort. However, recent results from the Aberdeen Children of the 1950s study suggests that childhood cognition is associated with harmful patterns of alcohol consumption, as indicated by self-reported symptoms of hangover (Batty et al., 2006). Since this association was in part mediated by adult SES, cognitive ability may be indirectly associated with safer patterns of alcohol consumption by facilitating entry into environments that promote good health management. Yet, higher adult SES was largely associated with high levels of alcohol consumption per se in the NSHD (Richards et al., 2005). In view of these findings, mental health outcomes in the present study were self-reported symptoms of anxiety and depression representing internalising disorder, and self-reported potential alcohol abuse representing externalising disorder.

The NSHD has obtained measures of early social circumstances, childhood cognitive ability, educational attainment, and prospective measures of adult SES, child and adult adverse circumstances, health behaviours, and mental health in midlife. In this birth cohort, early adverse circumstances, such as parental divorce, poor parental management, and poor material home conditions, are associated with relatively poor cognitive development (Richards & Wadsworth, 2004) and lower educational attainment (Ely, Richards, & Wadsworth, 1999; Richards & Wadsworth, 2004). Parental divorce in childhood and adolescence is also associated with affective disorder in the NSHD (Kuh & Hardy, 2002; Rodgers, 1990, 1994).

In a series of analyses, we examined whether childhood cognitive ability was associated with two mental health outcomes in mid-life, the 28 item General Health Questionnaire (GHQ-28) as a measure of internalising symptoms of anxiety and depression, and the CAGE screen for potential alcohol abuse as an externalising disorder. We then investigated whether any associations were explained by educational attainment, early disadvantage, or adult adverse circumstances, and adult health behaviours.

## Methods

### Study participants

Participants were drawn from the MRC NSHD), a birth cohort study stratified by social class and initially consisting of 5362 people selected from all births that occurred in England, Scotland, and Wales during one week in March 1946 (Wadsworth et al., 2003). Information about socio-demographic factors and medical, cognitive and psychological function has been repeatedly obtained by interview and examination. Most recently in 1999 at age 53 years, the sample size was reduced to 3035 based on loss to follow-up from the exclusion of deaths, persons living abroad or untraced, and permanent refusals. At this time the cohort was shown still to be a representative sample, in most respects, of the UK population legitimately and singly born in the immediate post-war era (Wadsworth et al., 2003). Of this sample, 1875 participants (918 males and 957 females) had completed data on all measures in this analysis. At age 53, 1160 of the 3035 cohort members interviewed had missing data for at least one variable in the analysis. Those not in the analysis were more likely to be male (*p*<0.001) and to have had significantly lower childhood cognitive ability (*p*<0.001) than those included in the analysis, although there was no difference at the 5% level in GHQ-28 score or in frequency of being CAGE positive at 53 years between those included and not included.

## Variables

*Mental health outcomes at 53 years*:

(a) Symptoms of anxiety and depression were assessed by the GHQ-28 (Goldberg & Hillier, 1979). The GHQ-28 is a self-administered screening questionnaire for detecting common mental disorder in the general population. Items on the questionnaire asked respondents if they had recent (over the past few weeks) complaints, such as lost much sleep over worry. Each individual item was scored using a 1–4 point Likert scale, and a total score was calculated (range 29–109; SD=9.56) and subjected to log transformation to improve the normality of the distribution.
(b) Potential alcohol abuse in the past year was assessed using the four CAGE questions (Ewing, 1984), i.e. C: Have you ever felt you ought to *cut down* on your drinking? A: Have people ever *annoyed* you by criticising your drinking? G: Have you ever felt bad or *guilty* about your drinking? E: Have you ever had a drink first thing in the morning to steady your nerves or to get rid of a hangover (*eye opener*)? Participants were divided into those scoring 0 or 1 vs. at least 2 on this measure, the latter indicating possible alcohol abuse as indicated by data presented by Ewing and others (see Ewing, 1984).

### Childhood cognitive ability

At 8 years survey members took four tests devised by the National Foundation for Educational Research (Pidgeon, 1964): reading comprehension (sentence completion), pronunciation, vocabulary and non-verbal reasoning. Raw scores for these tests were summed to obtain an overall score representing general cognitive ability. This score was normally distributed within the present study sample.

### Educational attainment

Educational attainment was based on the highest educational qualifications and their training equivalents attained by 26 years and were classified as none, vocational only, ordinary secondary (O levels), advanced secondary (A levels), or degree level or equivalent.

### Early circumstances and social stressors

The following variables represented early circumstances:

(a) SES of origin was represented by father's social class at age 11 or, if unknown, age 4 years or 15 years, and mother's education. Father's social class was classified as professional, managerial, intermediate, skilled manual, semi-skilled manual, or unskilled, according to the Registrar General, and dichotomised as non-manual vs. manual. Mother's education was indicated by four categories each for primary and secondary schooling levels (primary or secondary school only, primary or secondary and no diploma, primary or secondary and tech/course diploma, primary or secondary and qualifications/degree). These categories were recoded into two response categories: 0=primary school only and 1=secondary school or any training/qualifications.
(b) Material home conditions: an aggregate variable representing material home conditions at 4 years of age comprised ratings by a health visitor of age, cleanliness and state of repair of the dwelling, number of people per room, and cleanliness and condition of clothing and shoes of the survey member. This total score was then categorised into very good, good, modest, or poor.
(c) Parental divorce represented the time period up to age 8 years. Relatively small numbers of survey members experienced parental divorce as children, so the indicator variable was dichotomised to none vs. any by eight years.
(d) Maternal management and understanding at age 4 years of the survey member. As with the material home conditions variables, this was rated during the home-based interview by a health visitor, and scored as among the best, average, or among the worst. Because of very small numbers in the worst category in the sample selected for analysis, the indicator variable was dichotomised to good vs. average/poor.

### Adult SES

Adult SES was determined by own current or most recent occupational social class at 43 years using the same Registrar General scheme as for social class of origin and dichotomised as non-manual vs. manual. Net household income represented income after deduction for income tax, national insurance, state benefits and other income sources, such as interest, but included contributions from other household members (e.g. children) at 43 years and was dichotomised into<£14,999 vs. ⩾£15,000.

### Adult stressors

The following adult stressors at either or both 43 and 53 years were investigated:

(a) Unemployment was defined as not working full- or part-time and not in paid work.
(b) Financial hardship was defined as having to go without things that were really needed over the past 12 months because of lack of money.

These items were recoded into no experience of the stressor at either 43 or 53 years, vs. experiencing the stressor at either time point, or both.

### Health behaviours

The following health behaviours at 53 years were incorporated into the model, as possible confounders of the mental health outcomes:

(a) Physical exercise was classified as none vs. any in the past four weeks.
(b) Cigarette smoking was classified as none, up to 20 cigarettes per day, or 20+ per day.

## Statistical analysis

Multiple regression was used to test the association between childhood cognitive ability and the GHQ-28. Following a lifecourse model, we entered cognitive ability first and then adjusted for variables in chronological order. The unadjusted association between cognitive ability and the GHQ-28 (Model 1) was examined first and then adjusted for educational attainment (Model 2), entered as a categorical variable, and then progressively adjusted for the remaining variables, as follows: Model 3 adjusted for early circumstances to test the possibility that cognitive ability acts as a marker for early circumstances in relationship to mental health. Model 4 further adjusted for adult occupational social class and total household income, testing the proposition that SES influences mental health, above and beyond any effects of childhood cognitive ability or educational attainment. Model 5 further adjusted for the adult stressors of unemployment and financial hardship, since it may be that these SES-related stressors account for variability in mental health. Finally, Model 6 further adjusted for adult health behaviours considered in this context as probable concurrent coping responses in the presence of the adult stressors and possibly obscuring true associations between childhood cognitive ability and mental health. Based on previous evidence showing an association between potential alcohol abuse and depression (Hasin, Goodwin, Stinson, & Grant, 2005), we include the CAGE at 53 years as a potential covariate in the fully adjusted model for the GHQ-28. Model 6 was repeated for the four GHQ-28 subscales. All models were repeated for the CAGE outcome, using logistic regression. For both sets of analyses, the Sex×Childhood cognitive ability interaction terms were initially entered to test possible sex differences in the effect of childhood cognitive ability on the outcomes, in view of women being more likely than men to report greater symptoms suggestive of affective disorders (Kessler, 2003; Weissman et al., 1993) and men more likely than women to exhibit externalising behaviours, such as substance abuse (Sacker & Wiggins, 2002; van Os et al., 1997).

## Results

### Descriptive statistics

Descriptive statistics for study variables are presented in Table 1. While there was no gender difference in mean of childhood cognitive ability score (*p*=0.17), there was a gender difference in mean GHQ-28 scores (*p*<0.001). In addition, there was no obvious difference for men in mean GHQ-28 score between those scoring at or below the median for the cognitive score at 8 years and those scoring above the median, whereas, women above the median showed lower GHQ-28 scores (not shown). In contrast, men and women who screened positive on the CAGE had higher childhood cognitive ability scores than those who screened negative, particularly so for women (not shown).

### Childhood cognitive ability and the GHQ-28

Multiple linear regression was then used to test these effects, adjusting for sex only, after checking the assumption of linearity. Main effects for childhood cognitive ability and sex were significant at the 5% level, as was the childhood cognitive ability×sex interaction term (*p*=0.03), following which sex-stratified analyses were conducted.

Table 2 shows sex-stratified regression coefficients representing mean difference in GHQ-28 score per point increase in age 8 cognitive score for the fully adjusted model (Model 6).

While childhood cognitive ability was not associated with the GHQ in men at any stage of this modelling, it was inversely associated with this outcome in women, i.e. fewer symptoms of depression and anxiety were reported by women with higher childhood cognitive scores. Because the strength of this association for both men (unadjusted regression coefficient=0.003 (−0.15, 0.16), *p*=0.97) and women (unadjusted regression coefficient=−0.27 (−0.46, −0.08), *p*=0.005) remained roughly constant during all stages of covariate adjustment, Models 1–5 were not shown.

### Other covariates and the GHQ-28

In the fully adjusted model, increasing educational attainment by age 26 was associated with higher GHQ-28 scores in men and women (*p* for trend <0.01 and <0.01 respectively). For men, there was an association between educational attainment at the level of advanced qualifications and higher education and the GHQ in comparison to the reference category, but the overall F-test was not significant at the 5% level. For women, there were relationships between all four categories of educational attainment and the GHQ in comparison to the reference category. With the exception of the strong effect of having experienced parental divorce in women, there were few notable associations between early life factors and the GHQ-28. However, among the adult indicators, strong effects of unemployment, financial hardship, and screening CAGE positive at 53 years were observed in men and women, and physical exercise was associated with lower GHQ-28 scores in women. The childhood cognitive ability×sex interaction was confirmed in this fully adjusted model (*p*=0.04).

### Childhood cognitive ability and GHQ-28 subscales

To explore further the independent association between childhood cognitive ability and the GHQ-28, in women, the fully adjusted model was then applied to each of the four separate scales of this measure (not shown). There was no association between childhood cognitive ability and scale A (somatic symptoms: regression coefficient=0.005 (−0.31, 0.32), *p*=0.98). However, independent associations between childhood cognitive ability and scales B (anxiety and insomnia: regression coefficient=−0.45 (−0.80, −0.09), *p*=0.02), C (social dysfunction: regression coefficient=−0.22 (−0.41, −0.03), *p*=0.02) and D (severe depression: regression coefficient=−0.56 (−0.85, 0.28), *p*< 0.001) were all significant at the 5% level, with the strongest effect with the latter scale. These effects for childhood cognitive ability were not significant at the 5% level for men for all four separate scales of the GHQ-28.

### Childhood cognitive ability and alcohol problems

The childhood cognitive ability×sex interaction for this second outcome, CAGE at 53 years, was not significant at the 5% level, (*p*=0.26), so these analyses were conducted on the whole sample. In the unadjusted model (Model 1), an increasing childhood cognitive ability score (per item increase) was significantly associated with increasing odds of screening positive (odds ratio= 1.05 (1.02, 1.07), *p*=0.001). The strength of this association remained constant at an odds ratio of 1.05 (*p*<.01) during all subsequent stages of covariate adjustment in Models 2–6 (not shown).

To check that this positive association was not due to response bias (e.g. those of higher cognitive ability being more likely to worry or feel guilty about alcohol consumption even when their actual level of intake was relatively low), analyses were repeated using above or below a self-reported level of 21 units of alcohol per week as the outcome. A similar pattern of results was observed.

Table 3 shows odds of screening positive on the CAGE per point increase in childhood cognitive ability score for complete data in the fully adjusted model (Model 6). With the exception of positive associations between income and the CAGE and smoking and the CAGE, there were few effects of the covariates on this outcome. Indeed, childhood cognitive ability was one of the only variables in the model to show such an association.

## Discussion

In this population-based birth cohort study, we found evidence of associations between childhood cognitive ability and mental health in midlife that were independent of a wide range of potential confounders and mediators. However, no simple conclusion can be drawn from these results. Childhood cognitive ability was inversely associated with symptoms of anxiety and depression, as measured by the GHQ-28, but in women only. On the other hand, higher childhood cognitive ability scores were associated with increased risk of potential alcohol abuse in men and women, as indicated by the CAGE screen.

There was disproportionate loss to follow-up among those of lower cognitive ability, although we have no reason to conclude that this affected the pattern of the associations found in this study. However, greater attrition among those with more affective symptoms and more problems with alcohol abuse may have resulted in an underestimation of the low childhood cognitive ability-GHQ-28 association and an overestimation of the high childhood cognitive ability-CAGE association. Despite this limitation, this study has several strengths, including the use of mental health outcome measures that have been validated in previous studies and the prospective, longitudinal design of this population survey containing detailed information about factors that potentially explain the proposed mechanisms linking childhood cognitive ability to mental health outcomes. With these limitations and strengths in mind, the present analysis revealed findings worthy of detailed consideration.

Higher childhood cognitive scores were associated with fewer symptoms of anxiety and depression in women. While this is consistent with previous studies in this cohort (van Os et al., 1997) and other samples (Batty et al., 2005; Feinstein & Bynner, 2004; Zammit et al., 2004), the present study extends these findings by showing that the effect is independent of plausible explanatory factors, i.e. educational attainment and early and adult SES and adversity, and was not confounded by health behaviours, considered in this context as possible coping mechanisms. However, the present study also complicates previous findings, since this effect was observed only in women. Applying the fully adjusted model to the four individual subscales of the GHQ-28 indicated that the strongest associations were with Anxiety and Insomnia and Severe Depression, arguably representing the core symptomatology of affective disorder in general population samples.

Consistent with the literature concerning sex differences in the experience of affective disorders, mean GHQ-28 scores were higher for women in comparison to men across all study variables. While men were more likely than women to have been excluded from the analyses because of missing data, drop-out bias seems unlikely to have accounted for the large gender difference in effect size for cognition and the GHQ-28.

The well-known increased risk of affective disorder in women (e.g. Kessler, 2003) may have some biological basis, since it is associated with changes in sex-steroid levels during puberty (Angold, Costello, Erkanli, & Worthman, 1999). However, this is unlikely to account for the negative association found in the British 1946 cohort between childhood cognitive ability and symptoms of anxiety and depression in women in mid-adulthood, for the following two reasons. First, early menarche is associated with increased risk of mental health problems (Kaltiala-Heino, Marttunen, Rantanen, & Rimpela, 2003), yet is associated with higher cognitive ability in the NSHD (Douglas & Ross, 1964). Second, while higher childhood cognitive ability is associated with later menopause in the NSHD (Kuh et al., 2005; Richards, Kuh, Hardy, & Wadsworth, 1999), menopausal status is not associated with psychological symptoms (Kuh, Hardy, Rodgers, & Wadsworth, 2002).

In the absence of any compelling evidence that the gender-specific effect has a biological basis, a more psychosocial-based explanation might be investigated. Since cognitive ability reflects an individual's ability to learn and adapt to social and environmental demands, it may provide a psychological coping mechanism, or source of resilience to adverse circumstances, that are of greater benefit to women. Further work is needed to explore this possibility.

Second, contrary to the protective effect of cognitive ability on affect, greater childhood cognitive ability was associated with *increased* risk of self-reported alcohol abuse. The effect size may appear to be modest, but we should note that it represents a 2.4 increase in risk for those with age 8 cognitive scores above the mean, with reference to those who scored below the mean. This potentially contradicts the findings of Batty et al. (2006), which showed that higher childhood cognitive ability was associated with lower frequency of self-reported hangover in the Aberdeen Children of the 1950s study. However, it is consistent with population-based evidence from the UK that alcohol consumption tends to be positively associated with SES, in this cohort (Richards et al., 2005) and elsewhere (Britton, Singh-Manoux, & Marmot, 2004). The association between SES and alcohol consumption may be explained by cognitive ability, since this variable was positively associated with alcohol intake, as well as with possible risk patterns of consumption reflected by the CAGE in this study.

Third, for a given cognitive ability score, greater educational attainment was associated with higher GHQ-28 scores, independently of all other study variables. The bivariate relationship between greater educational attainment and higher GHQ-28 scores was not significant at the 5% level for men or women. Thus, higher GHQ-28 was associated with higher education only in the fully adjusted model with childhood cognitive ability. Contrary to the notion that education provides protective resources and a sense of control in one's life (Mirowsky & Ross, 2003), such benefits were not evident in midlife in this cohort. It is possible that we are seeing important differences in the process through which ability (more related to cognitive ability) vs. achievement (better represented by educational attainment) impact mental health. In this cohort of women, childhood cognitive ability may better reflect the ability to solve problems, apply skills, and utilise coping resources to protect against symptoms of internalising disorders. Finding those with higher educational attainment to have greater number of symptoms of anxiety and depression, on the other hand, having controlled for childhood cognitive ability may reflect role strain (Pearlin, 1983) or role overload (Heiss, 1992) in this cohort, characterised by working and living in environments that are more demanding than the ability to cope.

The divergent effects of education and childhood cognitive ability on the GHQ-28 were unexpected. Given the evidence of a strong interrelationship between education and cognition (e.g. Richards & Sacker, 2003; Rutter, 1985), the prior entry of cognitive ability may have been a suppressor due to overlapping variance with education. Other studies may find that cognitive ability makes a small contribution to health when education is entered first in the equation. Further research is needed before drawing any conclusions about the positive relation between education and distress.

To conclude, for women, childhood cognitive ability may reflect the skills and ability needed to protect against greater anxiety and depression in adulthood. It is also possible that greater cognitive ability may lead women into more protective environments. In contrast, future work is needed to explore why greater childhood cognitive ability places both men and women at higher risk for potential alcohol abuse. Investigations into the early manifestations of externalising mental health symptoms during developmental years and attempts to elaborate possible psychosocial mechanisms that may be associated with both childhood cognitive ability and greater risk for alcohol abuse need further consideration.

## Figures and Tables

**Table 1:** Descriptive statistics for all study variables: top panel presents means (sd) and bottom panel *N* (%)

	Males (*N*=918)	Females (*N*=957)	All (*N*=1875)
GHQ-28 at 53 years (range=28 to 112)	43.33 (8.01)	47.12 (10.63) *p*<0.001	45.26 (9.62)
Childhood cognitive ability at age 8 (range=0 to 45)	24.44 (6.86)	22.87 (6.71) *p*=0.17	22.66 (6.79)
*CAGE at 53 years*			
Negative	91.2 (837)	94.1 (901)	92.7 (1738)
Positive	8.8 (81)	5.9 (56)	7.3 (137)
*Educational attainment by 26 years*			
No qualifications	34.0 (312)	33.2 (318)	33.6 (630)
Vocational	5.6 (51)	10.4 (1 0 0)	8.1 (151)
Ordinary (‘O’ level)	15.6 (143)	26.4 (253)	21.1 (396)
Advanced (‘A’ level)	29.5 (271)	24.1 (231)	26.8 (502)
Higher	15.4 (141)	5.7 (54)	**10.5 (196) **
*Father's occupational social class*			
Manual	56.4 (518)	58.8 (563)	57.7 (1081)
Non-manual	43.6 (400)	41.2 (394)	42.3 (794)
*Mother's education*			
No qualifications	59.3 (544)	63.6 (609)	61.5 (1153)
Qualifications	40.7 (374)	36.4 (348)	38.5 (722)
*Parental divorce by age 8 years*			
No	97.1 (891)	97.1 (929)	97.1 (1820)
Yes	2.9 (27)	2.9 (28)	2.9 (55)
*Material home conditions at 4 years*			
Very good	24.3 (223)	28.2 (270)	26.3 (493)
Good	41.2 (378)	41.4 (396)	41.3 (774)
Modest	27.5 (251)	24.6 (235)	26.0 (487)
Poor	7.1 (65)	5.9 (56)	6.5 (121)
*Maternal management and understanding*			
Good	51.5 (473)	53.1 (508)	52.3 (981)
Average/poor	48.5 (445)	46.9 (449)	47.7 (894)
*Adult occupational social class by 43 years*			
Manual	37.0 (340)	25.5 (244)	31.1 (584)
Non-manual	63.0 (578)	74.5 (713)	68.9 (1291)
*Net household income at 43 years*			
<£14,999 per year	46.8 (430)	51.0 (488)	49.0 (918)
⩾ £ 15,000 per year	53.2 (488)	49.0 (469)	51.0 (957)
*Economic hardship 43 and/or 53 years*			
No	83.4 (766)	80.5 (770)	81.9 (1536)
Yes	16.6 (152)	19.5 (187)	18.1 (339)
*Unemployment 43 and/or 53 years*			
No	85.4 (784)	68.2 (653)	76.6 (1437)
Yes	14.6 (134)	31.8 (304)	23.4 (438)
*Physical exercise at 53 years*			
None	54.4 (499)	52.0 (498)	53.2 (997)
Any	45.6 (419)	48.0 (459)	46.8 (878)
*Cigarette smoking at 53 years*			
None	78.2 (718)	78.2 (748)	78.2 (1466)
0–20	15.4 (141)	20.09 (191)	17.7 (332)
21+	6.4 (59)	1.9 (18)	4.1 (77)

**Table 2:** Full model estimates (regression coefficients and 95% confidence intervals) representing mean change in GHQ-28 scores at 53 years

		Males (*N*=918)			Females (*N*=957)		
		Regression coefficient	(95% CI)	*p*	Regression coefficient	(95% CI)	*p*
Childhood cognitive ability at 8 years^a^ (per point increase)		−0.04	(−0.22, 0.15)	0.70	−0.29	(−0.52, −0.06)	0.01
*Education by 26 years*^b^*(ref.=no qualifications)*				0.11			0.02
Vocational only		2.04	(−2.72, 6.80)		5.81	(1.37, 10.26)	
Ordinary (‘O’ level)		2.57	(−0.88, 6.01)		5.59	(1.92, 9.26)	
Advanced (‘A’ level)		4.03	(0.94, 7.13)		5.04	(0.80, 9.29)	
Higher		4.92	(0.70, 9.13)		6.64	(−0.03, 13.30)	
Father's occupational social class (ref.=non-manual)		−1.32	(−3.80, 1.15)	0.29	−0.72	(−3.71, 2.27)	0.64
*Mother's education (ref.=no qualifications)*		−0.54	(−3.03, 1.95)	0.67	2.75	(−0.17, 5.67)	0.07
Parental divorce by 8 years (ref.=none)		−5.76	(−11.90, 0.39)	0.07	13.58	(6.16, 21.00)	<0.001
Material home conditions at 4 years^c^ (ref=very good)				0.55			0.38
Good		−0.12	(−2.81, 2.57)		1.13	(−1.98, 4.24)	
Modest		1.65	(−1.58, 4.89)		−1.14	(−4.92, 2.64)	
Poor		2.20	(−2.70, 7.11)		2.94	(−3.12, 9.00)	
Maternal management at 4 years (ref.=good)		−0.97	(−3.40 1.45)	0.43	0.75	(−2.05, 3.55)	0.60
Adult occupational social class (ref.=non-manual)		0.73	(−1.92, 3.37)	0.59	2.84	(−0.44, 6.13)	0.09
Household income at 43 years (ref.= <£14,999 per year)		−0.47	(−2.77, 1.84)	0.69	−1.81	(−4.47, 0.84)	0.18
Economic hardship 43 and/or 53 years (ref.=no)		9.64	(6.70, 12.57)	<0.001	6.08	(2.84, 9.34)	<0.001
Unemployment 43 and/or 53 years (ref.=no)		5.02	(1.99, 8.05)	0.001	4.04	(1.34, 6.73)	0.003
Physical exercise at 53 years (ref.=none)		0.30	(−1.89, 2.49)	0.79	−2.91	(−5.54, −0.27)	0.03
Smoking at 53 years (per level)		−0.58	(−2.45, 1.30)	0.55	2.16	(−0.63, 4.95)	0.13
CAGE positive by 43 years (ref.=no)		5.65	(1.99, 9.31)	0.003	10.04	(4.68, 15.39)	<0.001

a The size of the unadjusted regression coefficients for men (.003 (−0.15, 0.16), *p*=0.97) and women (−0.27 (−0.46, −0.08), *p*=0.005) in Model 1 were minimally affected by subsequent stages of covariate adjustment in Models 2–5.

b For men, trend for linearity *p*<0.01; for women, *p*<0.01; *p-*value given for ANOVA comparing means.

c For men, trend for linearity *p*=0.25; for women, *p*=0.80; *p-*value given for ANOVA comparing means.

**Table 3:** Full model estimates (odds ratios and 95% confidence intervals) representing the risk of screening positive for the CAGE screen at 53 years

		.All (*N*=1875)		
		Odds ratio	(95% CI)	*p*
?Childhood cognitive ability at 8 years (per point increase)^a^		1.04	(1.01, 1.07)	0.02
Sex (ref.=male)		0.11	(0.07, 0.18)	<.001
*Education by 26 years*^b^*(ref.=no qualifications)*				
Vocational only		0.67	(0.25, 1.80)	0.43
Ordinary (‘O’ level)		1.79	(1.02, 3.13)	0.04
Advanced (‘A’ level)		1.83	(1.07, 3.14)	0.03
Higher		1.58	(0.78, 3.21)	0.21
Father's occupational social class (ref.=non-manual)		0.88	(0.59, 1.31)	0.53
Mother's education (ref.=no qualifications)		0.71	(0.47, 1.06)	0.09
Parental divorce by 8 years (ref.=none)		1.79	(0.74, 4.31)	0.20
*Material home conditions at 4 years*^b^ (ref.=very good)				
Good		0.79	(0.51, 1.21)	0.28
Modest		1.01	(0.60, 1.70)	0.98
Poor		1.48	(0.67, 3.26)	0.33
Maternal management at 4 years (ref.=good)		1.02	(0.68, 1.51)	0.94
Adult occupational social class (ref.=non-manual)		1.04	(0.65, 1.65)	0.87
Household income at 43 years (ref.=<£14,999 per year)		1.56	(1.06, 2.30)	0.03
Economic hardship 43 and/or 53 years (ref.=no)		0.59	(0.34, 1.03)	0.06
Unemployment 43 and/or 53 years (ref.=no)		1.48	(0.95, 2.33)	0.09
Physical exercise at 53 years (ref.=none)		1.24	(0.86, 1.78)	0.25
Smoking at 53 years (per level)		1.64	(1.23, 2.19)	0.0001

a Unadjusted odds ratio was 1.05 (1.02, 1.07), *p*=.001, and the odds ratio remained the same in Models 2-5 after all subsequent covariate adjustments.

b Trend for linearity ns.
